# Impact of lordotic cages in the restoration of spinopelvic parameters after dorsal lumbar interbody fusion: a retrospective case control study

**DOI:** 10.1007/s00264-020-04719-2

**Published:** 2020-07-13

**Authors:** Stavros Oikonomidis, Vincent Heck, Sonja Bantle, Max Joseph Scheyerer, Christoph Hofstetter, Stefan Budde, Peer Eysel, Jan Bredow

**Affiliations:** 1 Faculty of Medicine and University Hospital Cologne, Department of Orthopedics and Trauma Surgery, University of Cologne, Kerpener Str. 62, 50937 Cologne, Germany; 2 Department of Neurological Surgery, University of Washington, Seattle, WA USA; 3 Department of Orthopedic Surgery, Hannover Medical School, Anna-von-Borries-Strasse 1-7, 30625 Hanover, Germany

**Keywords:** Spinal fusion, Interbody fusion, Cage, Lordotic angle, Sagittal balance, Lumbar lordosis

## Abstract

**Purpose:**

Aim of this study was to compare the reconstruction of radiological sagittal spinopelvic parameters between lordotic (10°) and normal cages (0°) after dorsal lumbar spondylodesis.

**Methods:**

This retrospective monocentric study included patients who received dorsal lumbar spondylodesis between January 2014 and December 2018. Inclusion criteria were degenerative lumbar diseases and mono- or bi-segmental fusions in the middle and lower lumbar region. Exclusion criteria were long-distance fusions (3 segments and more) and infectious and tumour-related diseases. The sagittal spinopelvine parameters (lumbar lordosis, segmental lordosis, sacral slope, pelvic incidence, and pelvic tilt) were measured pre- and post-operatively by two examiners at two different times. The patients were divided into 2 groups (group 1: lordotic cage, group 2: normal cage).

**Results:**

One hundred thirty-eight patients (77 female, 61 male) with an average age of 66.6 ± 11.2 years (min.: 26, max.: 90) were included in the study based on the inclusion criteria. Ninety-two patients (66.7%) received 0° cages and 46 (33.3%) lordotic cages (10°). Segmental lordosis was increased by 4.2° on average in group 1 and by 6.5° in group 2 (*p* = 0.074). Average lumbar lordosis was increased by 2.1° in group 1 and by 0.6° in group 2 (*p* = 0.378). There was no significant difference in the correction of sagittal spinopelvic parameters. Inter- and inter-class reliability was between 0.887 and 0.956.

**Conclusion:**

According to the results of our study, no advantages regarding sagittal radiological parameters for the implantation of a lordotic cage could be demonstrated.

## Introduction

Lumbar and lumbosacral spinal fusion is an established surgical procedure for the treatment of several degenerative conditions and deformities of the lumbar spine [1]. Many surgical techniques have been described in order to achieve fusion [1, 2]. Pedicle screw–based lumbar spinal fusion can sufficiently stabilise and restore the anatomy and alignment of the operated segments. Posterior and transforaminal lumbar interbody fusion (PLIF/TLIF) are common surgical procedures, and several studies report sufficient long-term clinical outcomes and low morbidity rates [3].

Restoration of the sagittal alignment of the lumbar spine can influence the clinical outcome after surgical treatment of degenerative spinal diseases [4]. Many studies postulate that restoring spinopelvic angulation leads to better clinical outcomes and additionally decreases the development of adjacent segment disease after lumbar spinal fusion [5, 6]. On the other hand, post-operative spinopelvic malalignment, referred to as a mismatch between pelvic incidence (PI) and lumbar lordosis (LL) of greater than 10°, leads to higher reoperation rates and reduced quality of life [7, 8]. Thus, restoring the spinopelvic alignment of the lumbar spine is considered to be an important goal of fusion surgery, besides solid fusion and decompression of the neural structures. The restoration of the balance between pelvic incidence and lumbar lordosis can be achieved by increasing segmental lordosis of the treated segment in case of fusion [9].

Surgical approaches to the lumbar spine and fusion techniques can have an influence on the restoration of the radiological spinopelvic parameters. Anterior approaches to the lumbar spine seem to allow a better restoration of segmental lordosis and, consequently, of the PI-LL mismatch than posterior approaches [2]. However, anterior surgical approaches can potentially lead to major approach-related complications, such as arterial and venous vascular injuries, retrograde ejaculation, and paralytic ileus [10]. PLIF and TLIF techniques can also restore segmental lordosis. Kepler et al. reported segmental increases in lordosis of up to 3.6–5.5° using a TLIF technique [11].

Besides the surgical approach and technique, only a few studies have reported about the influence of cage geometry in the restoration of segmental lordosis [12]. According to a finite element study of Uribe et al., hyperlordotic cages (20° and 30°) with anterior longitudinal ligament release can significantly increase segmental lordosis as compared with 10° lordotic cages [13]. Some clinical studies have also reported significant increases in segmental lordosis when using lordotic cages as compared with non-lordotic cages [12, 14, 15]. However, all these clinical studies included and compared small samples.

The aim of this study was to analyse and compare the restoration of the radiological spinopelvic sagittal parameters of the lumbar spine after mono- or bi-segmental or three segmental posterior and transforaminal lumbar interbody fusion using lordotic (10°) and non-lordotic (0°) cages in a large patient collective. The wedged design of lordotic cages could help restore segmental lordosis. The hypothesis of the study was that lordotic cages can increase segmental lordosis of the operated segment and consequently restore lumbar lordosis and spinopelvic alignment.

## Materials and methods

### Study design

We conducted a monocentric retrospective case control study. We enrolled all consecutive patients who received dorsal mono- and bi-segmental or three segmental lumbar interbody fusion in our institution because of symptomatic degenerative lumbar diseases or deformities in the middle and lower lumbar region (L3-S1) between 01 January 2014 and 31 December 2018. Exclusion criteria were long segmental fusions (four segments or more), infectious disease, vertebral fractures, severe osteoporosis or tumour-related diseases. All patients underwent lumbar spinal interbody fusion using posterior or transforaminal lumbar spinal fusion techniques (PLIF/TLIF). All procedures were performed by three senior orthopaedic spine surgeons. During the study period, one surgeon preferentially used lordotic cages while the other two surgeons mainly used non-lordotic cages.

**Fig. 1:**
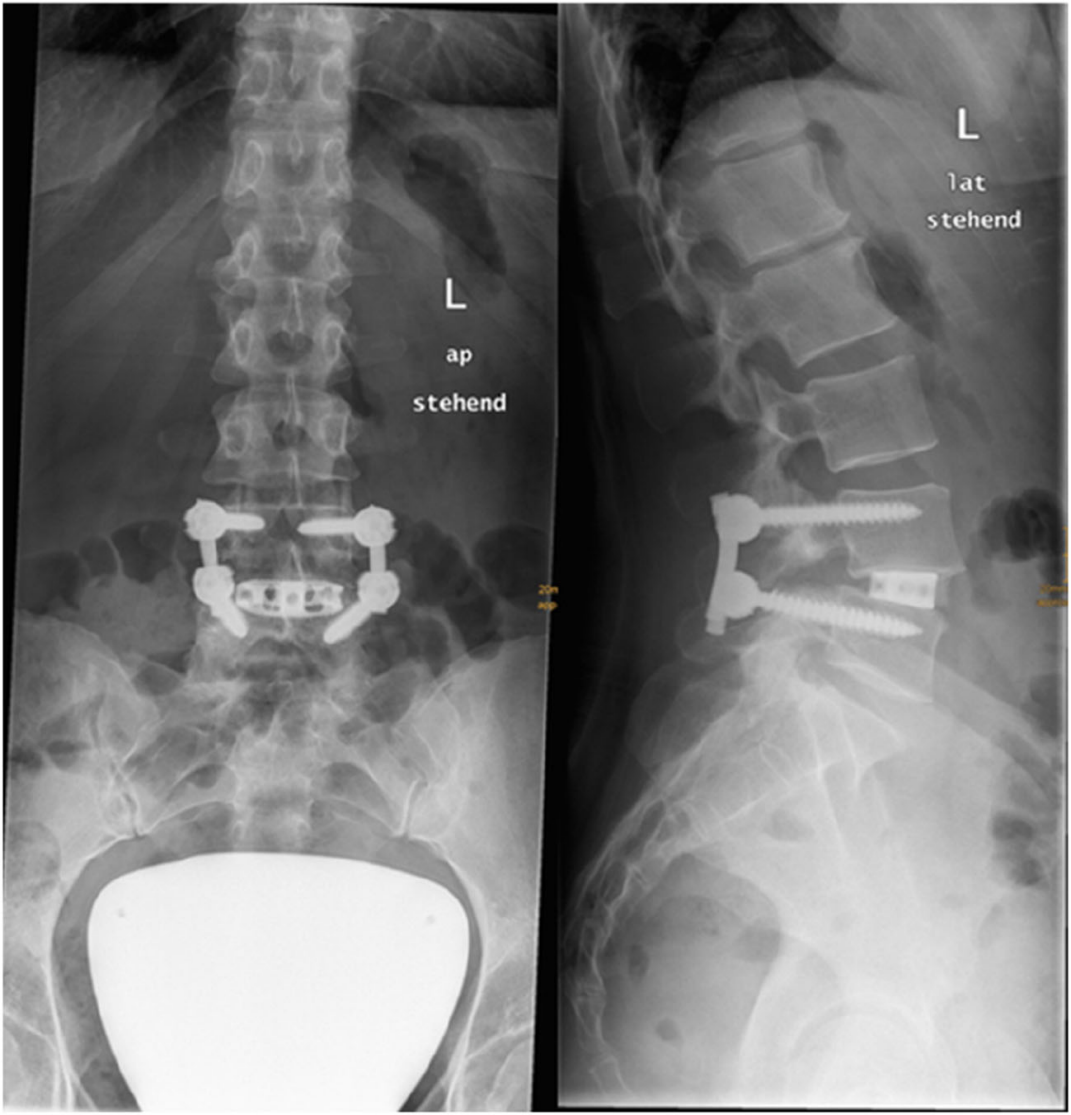
Example of X-rays (anterior-posterior and lateral) of a dorsal lumbar fusion L4/5 with a 10° lordotic cage (group 1)

**Fig. 2:**
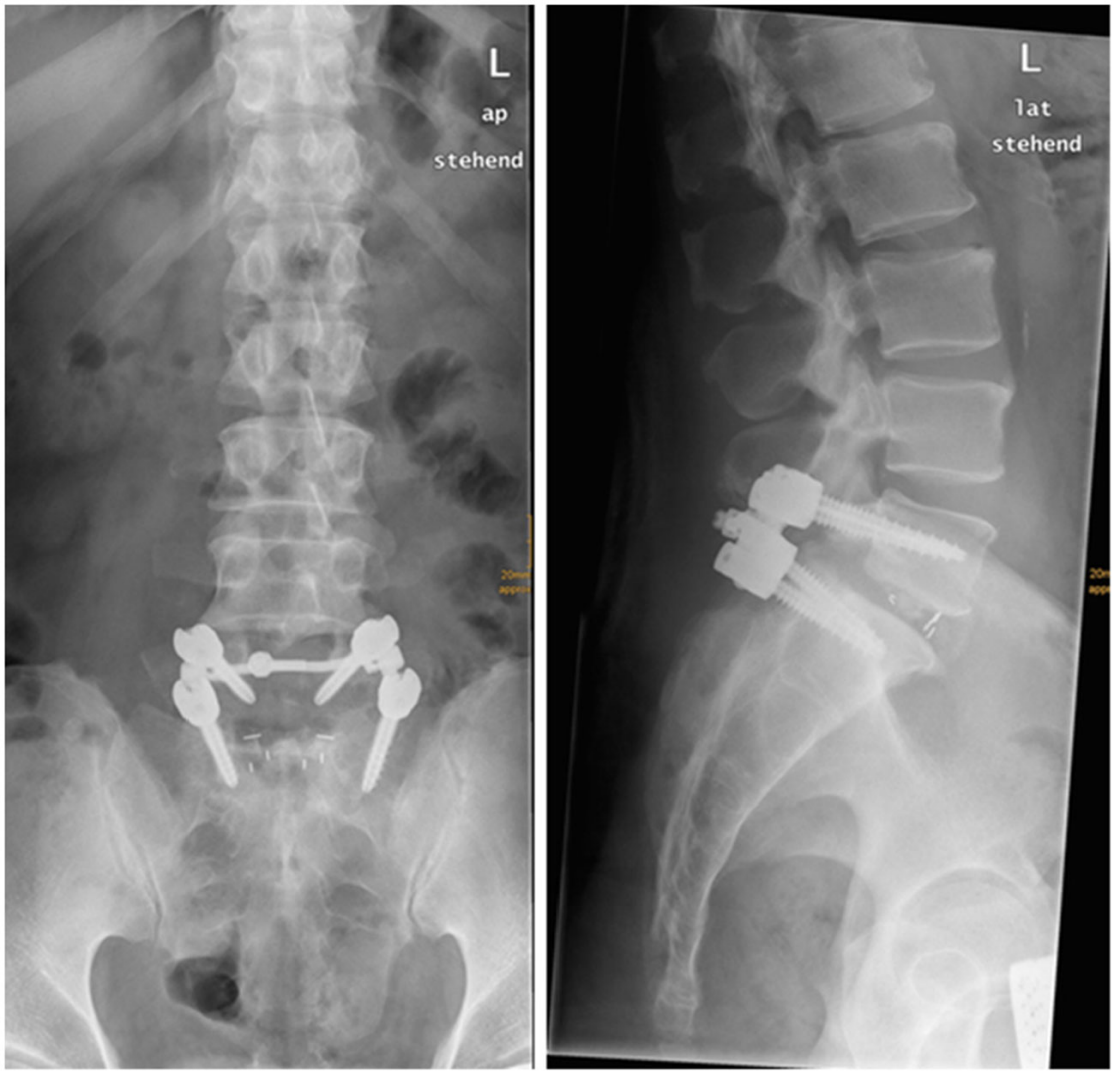
Example of X-rays (anterior-posterior and lateral) of a dorsal lumbar fusion L5/S1 with a 0° cage (group 2, control group)

The included patients were divided into 2 groups (group 1 (see Fig. 1): lordotic cage (10°) in TLIF technique; group 2, control group (see Fig. 2): non-lordotic cage (0°) in PLIF technique).

### Surgical techniques

All patients were operated in the prone position. After midline incision and exposure of the landmarks, pedicle screw insertion was performed using fluoroscopy. Standard titanium multiaxial pedicle screws were used in all patients. After screw insertion, posterior decompression by subtotal laminectomy and bilateral facetectomy were performed in case of posterior lumbar interbody fusion. After thorough discectomy and endplate preparation, the cages were inserted, and their position was controlled using fluoroscopy in the lateral and anterior-posterior view. In the non-lordotic group, a posterior lumbar interbody fusion was performed in all cases. PEEK cages (CAPSTONE® PEEK, Spinal System, Medtronic, Dublin, Ireland) were used in the non-lordotic group.

In the lordotic group, a transforaminal interbody fusion was performed. After unilateral facetectomy and laminotomy, a 10° lordotic titanium cage (ROCCIA® Multi-LIF Cage, Silony medical, Leinfelden-Echterdingen, Germany) was implanted. The position of the cage was controlled using fluoroscopy in the lateral and posterior anterior view.

### Data collection

#### Clinical data

Demographic data, such as gender, age and body mass index (BMI), and data regarding operation time were collected from the electronic data of the patients.

#### Radiological outcome measures

The radiographs of the lumbar spine in the anterior-posterior and lateral view in standing position pre- and post-operatively (3–5 days after surgery) were evaluated. The following sagittal spinopelvic parameters were measured by two experienced examiners at two different times: lumbar lordosis (the angle between the tangent lines to the cranial endplate of L1 and the cranial endplate of S1), segmental lordosis (the angle between the tangent line to the cranial endplate of the upper vertebra of the fusion and the caudal endplate of the lower vertebra of the fusion), sacral slope, pelvic tilt, and pelvic incidence. The two orthopaedic spine surgeons measured all the parameters using basic functions in IMPAX EE (Agfa HealthCare GmbH, Bonn, Germany). Intra- and inter-examiner reliability were measured by intra-class correlation. The pre- and post-operative radiological parameters were evaluated and compared between the two groups. Moreover, the difference between the pre- and postoperative radiological parameters was calculated in order to determine the correction.

#### Statistical analysis

The statistical analysis was performed using SPSS software (IBM SPSS Statistics version 25, 76 Chicago, IL, USA). Descriptive and frequency analyses were used to describe the demographic data, clinical data, and radiological outcomes. The Student *t* test for independent values was performed in order to analyse the radiological and clinical parameters between both groups and the Student *t* test for dependent samples for analysis of the radiological parameters within the groups. All reported *p* values have a two-tailed significance level of alpha = 0.05. No adjustment for multiple testing was performed.

Intra- and inter-examiner reliability were measured by intra-class correlation. Intra-class correlation coefficient (ICC) values were assessed in a two-way mixed model with absolute agreement at 95% confidence intervals for inter-observer reliability. Values < 0.40 were considered poor, those between 0.40 and 0.59 were considered fair, those from 0.60 to 0.74 were considered good, and those between 0.75 and 1.00 were considered excellent [16].

## Results

### Demographics and clinical data

One hundred thirty-eight patients (77 female, 61 male) with an average age of 66.6 years ± 11.2 (min.: 26, max.: 90) were enrolled in the study based on the inclusion criteria. The average body mass index was 30.1 kg/m^2^ ± 6.8 (min.: 18.8, max.: 57.7). The mean operation time was 162.5 minutes ± 45.7 (min.: 64, max.: 310). Seven patients (5.1%) were ASA 1, 69 patients (50.0%) ASA 2, 60 patients (43.5%) ASA 3, and two patients (1.4%) ASA 4. Ninety-six patients (69.6%) received a mono-segmental interbody fusion, 35 patients (25.4%) received a bi-segmental interbody fusion, and 7 patients (5.1%) received three segment interbody fusion.

In 46 patients (33.3%), a 10° lordotic cage (group 1) was used, and in 92 patients (66.7%), a 0° non-lordotic cage (group 2) was implanted. In Table 1, the demographical and clinical data of the included patients are compared between the two groups. No statistically significant differences could be verified in the demographic and clinical data between the two groups.

**Table 1:** Statistical analysis of the demographics and clinical data in group 1 (lordotic cage) and group 2 (non-lordotic cage)

		Group 1		Group 2		
		*N*	Mean value	*N*	Mean value	*p* value
Sex	Male	23		38		0.332
	Female	23		54		
Age			66.3 ± 10.3 (range, 44–85)		66.7 ± 11.6 (range, 26–90)	0.832
Segments			1.43 ± 0.6		1.32 ± 0.6	0.253
ASA classification	ASA 1	2		5		0.956
	ASA 2	23		46		
	ASA 3	20		40		
	ASA 4	1		1		
Operation time			154.5 ± 47.1		166.5 ± 44.7	0.145
BMI			30.4 ± 7.1 (range, 20.4–50.1)		29.9 ± 6.7 (range, 18.8–57.7)	0.720

### Radiological outcome

In both groups, segmental lordosis was significantly increased post-operatively (group 1: preo-perative: 21.7°, post-operative: 25.9°, *p* = 0.000; group 2: pre-operative: 16.8°, post-operative: 23.3°, *p* = 0.000). Regarding lumbar lordosis, an increase could be observed in both groups; however, this was without any statistical significance (group 1: pre-operative: 45.1°, post-operative: 46.7°, *Δ* = 1.6°, *p* = 0.651; group 2: pre-operative: 43.8°, post-operative: 45.9°, *Δ* = 2.1°, *p* = 0.060) (see Table 2).

**Table 2:** Descriptive statistical analysis of the radiological parameters pre- and postoperatively in group 1 (lordotic cage) and group 2 (non-lordotic cage). The mean values of the two groups have been compared using the Student *t* test for independent samples and within the groups, the *t* test for paired samples

Radiological parameters in °		Group 1	Group 2	*p* value
Lumbar lordosis	Pre-operatively	45.1 ± 15.3	43.8 ± 15.1	0.651
	Post-operatively	46.7 ± 12.5	45.9 ± 11.7	0.908
	*p* value^•^	0.651	0.060	
Segmental lordosis	Pre-operatively	21.7 ± 10.1	16.8 ± 10.3	0.009*
	Post-operatively	25.9 ± 10.1	23.3 ± 10.1	0.167
	*p* value^•^	0.000*	0.000*	
Sacral slope	Pre-operatively	33.9 ± 11.6	33.3 ± 10.2	0.738
	Post-operatively	33.6 ± 10.3	34.8 ± 8.9	0.492
	*p* value^•^	0.773	0.058	
Pelvic tilt	Pre-operatively	55.6 ± 15.8	58.5 ± 14.9	0.302
	Post-operatively	55.4 ± 15.2	58.8 ± 14.3	0.207
	*p* value^•^	0.838	0.767	
Pelvic incidence	Pre-operatively	23.4 ± 11.0	27.0 ± 11.1	0.073
	Post-operatively	24.2 ± 9.9	26.0 ± 10.3	0.331
	*p* value^•^	0.409	0.302	

*Statistical significance

^•^*p* value between pre- and post-operative parameters

The pre- and post-operative radiological data were compared between the two groups. Regarding the mean pre-operative spinopelvic parameters, lumbar lordosis, sacral slope, pelvic tilt, and pelvic incidence were comparable between the two groups. However, in group 2, pre-operative segmental lordosis was significantly lower than in group 1 (group 1: 21.7°, group 2: 16.8°, *p* = 0.009). The post-operative sagittal radiological parameters were similar in both groups and did not show any significant differences. The detailed pre- and post-operative radiological sagittal parameters are presented in Table 2.

Regarding the correction of the sagittal radiological parameters, no significant differences could be observed between the two groups. The correction in both segmental and lumbar lordosis was higher in group 2; however, this was without any statistical significance. Segmental lordosis was increased by 4.2° in group 1 and 6.5° in group 2, on average. Lumbar lordosis was also increased by 0.6° in group 1 and 2.1° in group 2, on average. Detailed data about the correction of the sagittal radiological parameters are presented in Table 3.

**Table 3:** Descriptive statistical analysis of the difference of the pre- and postoperative radiological parameters in group 1 (lordotic cage) and group 2 (non-lordotic cage). The mean values of the two groups have been compared using the Student *t* test for independent samples

Difference in the pre- and post-operative radiological parameters	Group 1	Group 2	*p* value
Δ Lumbar lordosis	− 0.6 ± 8.7	− 2.1 ± 10.5	0.406
Δ Segmental lordosis	− 4.2 ± 6.7	− 6.5 ± 7.7	0.087
Δ Sacral slope	0.3 ± 6.9	− 1.5 ± 7.6	0.175
Δ Pelvic tilt	0.2 ± 7.5	− 0.3 ± 9.0	0.743
Δ Pelvic incidence	− 0.9 ± 7.0	1.0 ± 9.0	0.229

Inter- and intra-class reliability was excellent, and ICC for the sagittal radiological parameters was between 0.887 and 0.956 [16].

## Discussion

The restoration of spinopelvic parameters is an important aim in lumbar spinal fusion. Loss of lumbar lordosis often leads to sagittal imbalance, adjacent segmental degeneration, and poor clinical outcomes [6, 17, 18]. PLIF and TLIF procedures are established methods of dorsal spinal fusion and can restore the sagittal alignment of the lumbar spine [11]. Biomechanical considerations suggest that greater postoperative lordosis can be achieved by using lordotic cages [19]. However, it remains unclear whether lordotic cages, in comparison with non-lordotic cages, provide additional benefits in radiological outcomes in clinical practice.

The main findings of this study suggest that the geometrical form of the cage used in cases of lumbar dorsal interbody fusion did not have an influence on the restoration of the sagittal radiological parameters. Both the 0° cages used in PLIF and the 10° cages used in TLIF procedures provide a significant improvement of segmental lordosis post-operatively. Lumbar lordosis could be increased in both groups; however, this was without any statistically significance. Interestingly, in our cohort, the segmental lordosis in group 2 (non-lordotic cage) was significantly lower than in group 1 (lordotic cage) pre-operatively (group 1: 21.7°, group 2: 16.8°, *p* = 0.009). However, the difference between pre- and post-operative segmental lordosis (Δ) did not reach statistical significance between the two groups. Thus, the results of our study indicate that both non-lordotic and lordotic cages lead to sufficient restoration of segmental lordosis. Few other studies have dealt with the influence of cage geometry on sagittal radiological parameters [12, 14, 15, 19].

Gödde et al. report that the use of lordotic cages has a significant influence on post-operative spinopelvic parameters [12]. The authors retrospectively examined 42 patients who had received dorsal instrumentation using PLIF, either with a lordotic or a non-lordotic cage. In the group with lordotic cages in segments L3/4 and L4/5, a 3° cage was used, and in segment L5/S1, an 8° cage was used. While post-operative lordosis was not significantly different between the two groups, segmental lordosis improved significantly in the group with lordotic cages in the merged segments. In the group of non-lordotic cages, segmental lordosis even decreased by 3 to 8°, whereby the authors attributed the cause of this difference to the geometry of the cage. Sembrano et al. also described a significant increase in post-operative lumbar lordosis after lateral lumbar interbody fusion using 10° lordotic cages, whereas non-lordotic cages had no significant influence on post-operative sagittal alignment (lordotic cages: *Δ* 2.8°, non-lordotic: *Δ* 0.6°) [15]. On the contrary, Dietrich et al. conducted a prospective radiographic study which included 40 patients undergoing mono-segmental fusion and compared the use of a 4° lordotic cage with a non-lordotic cage. Post-operatively, an improvement in lumbar lordosis was achieved in both groups, but there was no significant difference between the lordotic and the non-lordotic cages. The greatest increase of segmental lordosis was seen in segment L4/5 after fusion with the 4° lordotic cage [19]. A further study, reporting no difference in the radiological outcome between non-lordotic and lordotic cages, has been published by Takahashi et al. The authors report a similar restoration of segmental lordosis after PLIF surgery using a 0° cage (horizontal cylinder) and a 3° lordotic cage (open box) [20]. In general, all reported studies included a small patient collective and the use of lordotic cages smaller up to 10°.

Hong et al. reported that 15° lordotic angle cages create a significantly higher post-operative lumbar lordosis in mono- or bi-segmental TLIF than 4° or 8° cages. They postulated the use of 15° lordotic cages in the lower lumbar spine in order to achieve as much segmental lordosis as possible [14]. However, the authors also discuss that the restoration of the segmental lordosis by dorsal approaches is limited because of the tight anterior longitudinal ligament resulting in a limitation in widening the interbody space. Thus, the height or geometry of the cage can influence only partially the restoration of segmental lordosis.

These findings are supported by finite element and biomechanical investigations. Uribe et al. investigated, in a finite element study, the restoration of segmental lordosis and disc height after lumbar spinal fusion in 19 different simulations. The authors considered, and imported into their simulations, the anterior longitudinal ligament and the posterior elements such as facet joints and spinous processes. They compared different scenarios of anterior and posterior releases (such as release of the anterior longitudinal ligament, facetectomy, and posterior column resection) and implantation of lordotic (10°) and hyperlordotic cages (20° and 30°). They concluded that posterior and anterior releases lead to greater increases of segmental lordosis [13].

In a biomechanical investigation report, Melikian et al. commented that while a non-lordotic and 10° lordotic cages have a minor effect on post-interventional gain in segmental lordosis, the insertion of a hyperlordotic cage results in a gain of *Δ* 10.6° (± 3.9). However, the release of the anterior longitudinal ligament, spinous process resection, facetectomy, and compression with pedicle screw-rod constructs have the largest impact on post-operative segmental lordosis, up to *Δ* 26° (± 8.6) [21].

In addition, the positioning of the cage seems to play an important role in the restoration of segmental lordosis. Anterior positioning of the cages in PLIF and TLIF techniques with short cages results in greater post-operative segmental lordosis [22, 23].

In this sense, according to the results of our study and literature review, the geometry of the cage alone cannot influence the restoration of segmental lordosis significantly. In general, restoration of segmental and lumbar lordosis can be achieved through different techniques in cases of dorsal lumbar interbody fusion. Proper posterior release, such as facetectomy and anterior positioning of the cage, could also help restore segmental lordosis after lumbar spinal fusion [19, 20]. Furthermore, proper intra-operative positioning of the patient, especially of the hips, is reported to influence the restoration of the sagittal alignment of the lumbar spine [24]. Moreover, appropriate rod contouring can also improve the sagittal alignment of the lumbar spine [25]. Thus, the restoration of the segmental sagittal alignment can be affected through different surgical techniques and, as the results of the current study reveal, the geometry of the interbody implant does not seem to have a major influence on the reconstruction of the sagittal alignment. In summary, proper reconstruction of the sagittal alignment of the lumbar spine should be performed in case of lumbar interbody fusion.

**Fig. 3:**
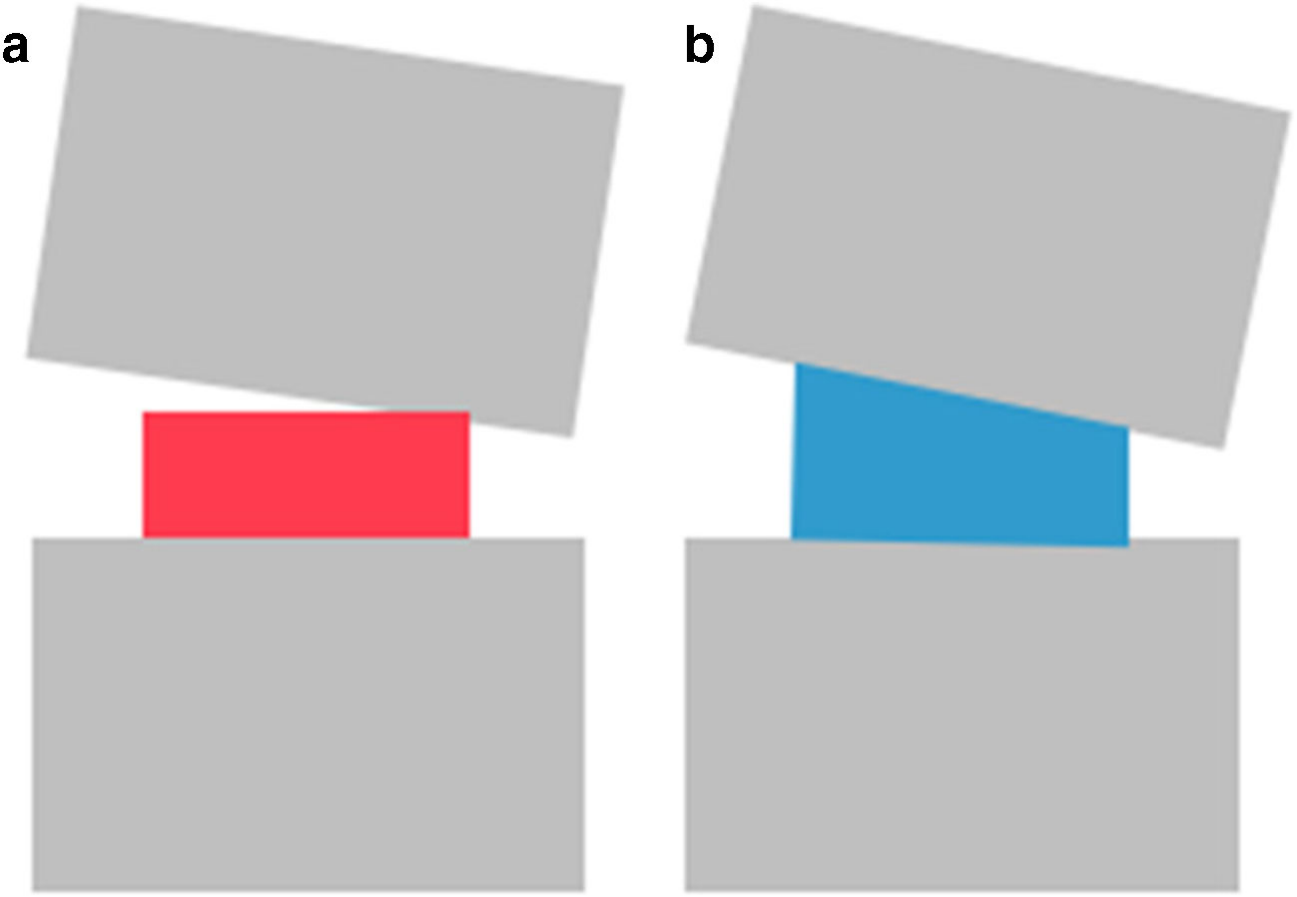
Illustration of the contact surface in cases of implantation of a non-lordotic (straight) interbody implant (**a**) and in cases of a lordotic (wedge-shaped) interbody implant (**b**) in a lordosis-stabilised segment. Lordotic cages provided a wider contact between the surface of the endplate and cage. This effect could reduce the pressure applied to the endplate of the segment

However, a possible advantage of lordotic cages could be the improved contact surface of the endplates in the fused segment. As the segment is stabilised in a lordotic angle, wedge-shaped cages could offer an extended contact surface as compared with non-lordotic cages. In case of straight (non-lordotic) cages, the contact surface of the endplates and the cage is limited when the segment is stabilised in a lordotic position (see Fig. 3). The wider contact surface of the wedge-shaped cages could reduce the pressure applied at the endplates preventing endplate failure and implant subsidence. A current biomechanical investigation has reported a decreased risk of endplate failure and implant subsidence in cases of implantation of self-adjusting, multiaxial end cap cages as compared with conventionally fixed angle cages [26]. This effect could reduce the incidence of pseudarthrosis and pedicle screw loosening in cases of lumbar interbody fusion. In addition, due to the improved contact surface of the endplates in the instrumented segment, the possible loss of segmental lordosis until spinal fusion is achieved could be lower when a lordotic cage is used. Clinical trials with a long-term follow-up comparing the two cage designs regarding the incidence of pseudarthrosis, endplate failure, and implant subsidence could provide useful evidence.

The current study has some limitations. Even though there is a large number of cases, it is a retrospectively designed study. As a consequence, randomisation was not possible. However, there is no statistical difference in patient demographics. The statistical power of this study is limited due to the retrospective study design. Because of the retrospective design of the study, no power analysis could be carried out in order to determine the sample size required to detect a significant difference between the two groups. Finally, the choice of the surgical procedure was solely determined by the preference of the surgeon and a performance bias of the surgeon could have an influence on the results.

## Conclusion

In short segment fusion, the post-operative spinopelvic parameters are not additionally improved by the use of lordotic cages. With both lordotic and non-lordotic cages, segmental lordosis is significantly increased. Other factors, such as manual repositioning of the screw-rod system or the extent of posterior element resection, are largely responsible for post-operative sagittal balance.
